# Animal models of methylmalonic acidemia: insights and challenges

**DOI:** 10.1186/s13023-025-04101-8

**Published:** 2025-11-14

**Authors:** Shan Shan, Min Liu, Yue Ma, Meng Sun, Yue Wang, Hui Zou

**Affiliations:** 1 Medical Research Center, Jinan Maternity and Child Care Hospital Affiliated to Shandong First Medical University, Jinan, 250000 China; 2 School of Clinical and Basic Medical Sciences, Shandong First Medical University & Shandong Academy of Medical Sciences, Jinan, 250117 China; 3 Newborn Disease Screening Center, Jinan Maternity and Child Care Hospital Affiliated to Shandong First Medical University, Jinan, 250000 China

**Keywords:** Methylmalonic acidemia, Animal models, Inherited metabolic diseases, Pathogenesis, Therapeutic strategies

## Abstract

Methylmalonic acidemia (MMA) is a rare genetic disorder caused by disruptions in the metabolism of methylmalonic acid, resulting in severe neurological and systemic complications. Animal models have become indispensable in advancing our understanding of MMA pathogenesis, evaluating treatment options, and exploring new therapies. This review highlights recent advancements in MMA research, focusing on the characteristics, advantages, and limitations of various genetically engineered animal models, including murine and zebrafish models. By integrating insights from these models, this work aims to provide a foundation for future clinical applications and therapeutic innovations.

**Clinical trial number** Not applicable.

Methylmalonic Acidemia (MMA), a rare autosomal recessive inherited metabolic disorder, is caused by enzyme deficiencies in the methylmalonic acid metabolic pathway [1, 2]. First reported in 1967, the disease’s pathogenesis primarily arises from mutations in genes encoding methylmalonyl-CoA mutase (MCM or MMUT) or proteins essential for synthesizing its coenzyme, 5’- deoxyadenosylcobalamin (a form of cobalamin) [3–5]. These mutations lead to reduced MCM activity, impairing the conversion of methylmalonyl-CoA to succinyl-CoA (critical intermediate in the tricarboxylic acid (TCA) cycle) and disrupting the oxidative metabolism of valine, isoleucine, methionine, threonine, cholesterol, and odd-chain fatty acids. Consequently, intermediate metabolites, such as methylmalonyl-CoA, methylmalonylcarnitine, methylmalonic acid, propionic acid, and propionylcarnitine (C3), accumulate in the blood, causing a spectrum of clinical symptoms in affected children. MMA typically manifests with systemic tissue damage, particularly affecting the central nervous system. Patients often experience recurrent acidosis, respiratory distress, delayed growth and development, and intellectual disabilities, lethargy and, in several cases, death [1–6]. The global prevalence of MMA varies significantly across regions, ranging from 1:50,000 to 1:360,000 live births, depending on geographic and population-specific factors [7, 8]. However, the prevalence in China is notably higher [9]. For instance, newborn screening data report incidence rates of 1:26,000 in Beijing and Shanghai, 1:3,920 in Shandong Province, and 1:6,032 in Henan Province [1].

In recent years, advancements in diagnostic technologies such as tandem mass spectrometry and gas chromatography-mass spectrometry have facilitated earlier diagnosis and management of MMA. Interventions such as low natural protein diets, carnitine supplementation, antibiotics to intermittently reduce propionate-producing gut bacteria, and organ transplantation (e.g., liver or liver/kidney) have significantly decreased mortality rates and delayed long-term complications [10, 11]. However, the acute-phase mortality and chronic multisystemic complications (particlularly neurological damage) remain significant challenges, severely impacting the quality of life of affected children.

## The pathogenesis of MMA

**Fig. 1:**
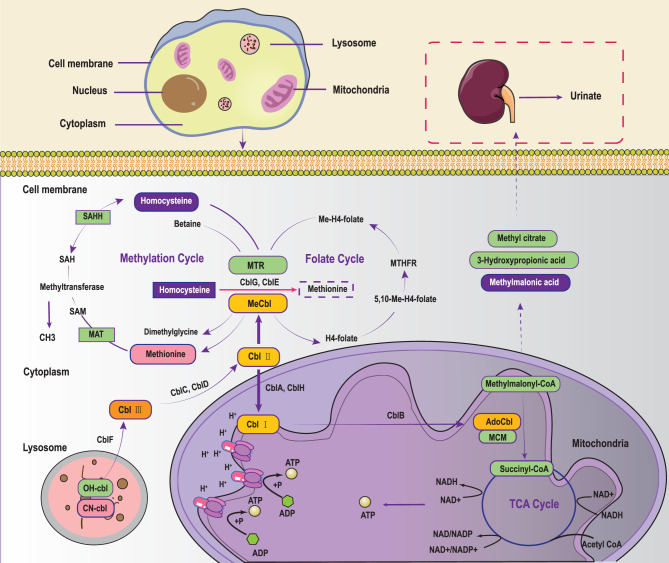
The cbl and MCM-dependent metabolic pathways. MTR: 5-methyltetrahydrofolate-homocysteine methyltransferase 5; MeCbl: Methylcobalamin; MAT: methionine Adenosyltransferase; SAM: S-adenosyl Methionine; SAH: S-Adenosyl-L-Homocysteine; SAHH: S-Adenosyl-L-Homocysteine Hydrolase; MTHFR: 5,10-methylenetetrahydrofolate Reductase; AdoCbl: Adenosylcobalamin; MCM: methylmalonyl-CoA mutase; cbl: cobalamin; TCA: tricarboxylic acid

Methylmalonic acid is a key metabolic intermediate involved in numerous biochemical reactions (See Fig. 1). Its primary metabolic pathway includes conversion to succinyl-CoA via MCM, which then participates in the TCA cycle and energy metabolism [3, 12]. Under normal conditions, methylmalonic acid concentrations are tightly regulated. However, in MMA patients, deficiencies in MCM or its coenzyme adenosylcobalamin (ado-cbl) lead to the accumulation of methylmalonic acid, resulting in metabolic disorders and various clinical symptoms [10]. MMA can be broadly categorized into two primary subtypes based on enzyme deficiencies: MCM deficiency (*mut*-type) and ado-cbl metabolism disorders. MCM deficiency arises from mutations in the *MMUT* gene and is further classified into *mut*^-^ and *mut*^0^ subtypes, depending on the presence or absence of residual MCM activity. The *mut*-type, accounting for approximately 30% of MMA cases, often leads to metabolic crises and neurological damage. The *MMUT* gene, located at 6p12-21.1, comprises 13 exons spanning a total length of 35kb, with over 250 mutations identified to date.

MMA caused by ado-cbl metabolism disorders includes several subtypes according the mutated genes of related enzyme: *cblA*, *cblB*, *cblC*, *cblD*, *cblD-2*, *cblF*, *cblJ*, and *cblX* subtypes [2, 10, 13–16]. *mut*^-^, *mut*^0^, *cblA*, *cblB*, and *cblD-2* subtypes are primarily characterized by elevated methylmalonic acid levels and are collectively referred to as isolated MMA [4, 17–19]. Conversely, the *cblC*, *cblD*, *cblF*, *cblJ* and *cblX* subtypes results in deficiencies in both ado-cbl and methylcobalamin synthesis. Ado-cbl serves as coenzyme for MCM, and its reduced activity increases methylmalonic acid levels. Similarly, methylcobalamin, a coenzyme for methionine synthase, is crucial for converting homocysteine to methionine. Its reduced activity leads to elevated homocysteine levels in the blood. Therefore, patients with *cblC*, *cblD*, *cblF*, *cblJ* and *cblX* subtypes exhibit methylmalonic acidemia combined with homocystinemia [2, 13, 16, 20, 21]. In China, combined MMA accounts for approximately 70% of cases, with the *cblC* type being the most prevalent due to mutations in the *MMACHC* gene [22, 23]. The *MMACHC* gene, located at 1p34.1, contains four exons and has over 100 pathogenic mutations documented in the human gene mutation database (HGMD, http://www.hgmd.cf.ac.uk/ac/index.php). Most of these mutations are missense and nonsense mutations. Among Chinese patients, the most frequently observed mutations in the *MMACHC* gene include *c.80A > G*, *c.609 G > A*, *c.482 G > A*, *c.394C > T*, and *c.658_660del* [24].

## Animal models in MMA studies

Understanding the pathogenesis of MMA has been challenging due to its complexity and variability. While clinical studies provide valuable insights, they are often limited by ethical consideratinos, small sample size, and the inability to explore invasive mechanistic pathways. As a result, preclinical models, particularly animal models, play a crucial role in bridging these gaps. These models provide opportunities to elucidate disease mechanisms, test therapeutic interventions, and serve as platforms for translational research.

The most commonly used animal for MMA research is *Mus musculus* (mouse), followed by *Rattus norvegicus* (rat), *Danio rerio* (zebrafish), *Caenorhabditis elegans* (worm) [25, 26], *Drosophila melanogaster* (fruit fly) [27], *Felis catus* (cat) [28], and *Canis familiaris* (dog) [29]. These models replicate key physiological and biochemical characteristics of human MMA, offering valuable platform for drug screening and therapeutic evaluation. Moreover, animal models enable researchers to investigate the effects and underlying mechanisms of methylmalonic acid on various systems, including the nervous and endocrine systems. Such studies are critical for understanding the systemic impacts of the disease. By using these models, scientists have gained insights into the pathophysiological mechanisms of MMA, paving the way for new directions in clinical treatment and therapy development.

One widely employed approach for creating MMA models involves the systematic or localized administration of organic acids, such as methylmalonate, to animals, including mice, rats, fruit flies, and zebrafish [27, 30–32]. While this method allows researchers to mimick the environmental changes associated with the disease, it represents only a single aspect of MMA pathophysiology and cannot fully capture the complexity of the disorder. Therefore, instead of focusing on these chemically indued models, this revies highlights genetically engineered models. These include models generated through gene knockouts, transgenesis, or the introductions of orthologous patient mutations via genome-editing technologies.

## Murine models of MMA

### *Mut*-type mouse models

Current global research on MMA primarily focuses on developing mouse models for the *mut*-type MMA caused by *MMUT* gene mutations, followed by models for the *cblC* type MMA associated with *MMACHC* gene mutations. *Mut*-type MMA arises from mutations in the *MMUT* gene, leading to MCM deficiency. Based on the residual activity of MCM, this condition is classified into two subtypes: the *mut*^0^ type, characterized by complete MCM deficiency, and the *mut*^-^ type, characterized by partial MCM deficiency.

Peters et al. were the first to use gene-targeting technology to disrupt the CoA-binding domain of MCM by replacing exon 3 of *MMUT* with an antibiotic selection marker, thereby creating a *mut*^0^ type MMA model referred to as the *Mut*^-/-^ mouse [33]. These mice appear normal at birth but completely lack MCM activity, resulting in rapid deterioration and death within 24 hours. Elevated levels of metabolites such as methylmalonic acid, methylcitric acids, and C3 are detectable in their blood and urine from birth, closely mimicking the severe phenotype observed in patients with *mut*^0^ type MMA [33, 34]. Using this murine model, Chandler et al. directly injected an E1/E3-deleted adenovirus containing murine *MMUT* cDNA into the livers of neonatal *Mut*^-/-^ mice. Treated mice exhibited reduced metabolite levels and extended survival compared to untreated controls [34]. Subsequent studies utilizing recombinant adeno-associated virus serotype 8 (rAAV8) vectors to deliver *MMUT* cDNA further demonstrated that treated mice could survive for at least one year [35]. These experiments provide preclinical evidences supporting the efficacy of gene therapy for MMA.

Given the neonatal or early lethality of *Mut*^-/-^ mice, which limits long-term studies Chandler et al. created an improved *Mut*^-/-^ mouse model in 2009 by introducing genes from the FVB/N strain into the (C57BL/6 × 129 Sv/Ev) *Mut*^+/-^ strain. This breeding approach produced a small fraction of triply mixed (C57BL/6 × 129 Sv/Ev×FVB/N) G2 *Mut*^-/-^ mice that survived beyond the neonatal period, exhibiting elevated methylmalonic acid levels in the blood. These mice allowed researchers to study the pathophysiology and therapeutic interventions for MMA until weaning [36].

Further advancements include the generation of transgenic mice carrying different copy numbers of the human MCM locus. In 2012, Peters et al. created four transgenic mouse lines and crossed a two-copy line with heterozygous knockout *MMUT* mice [33] This cross produced hemizygous mice (*Mut*^-/-^;*MUT*^2h^) with partial rescue of neonatal lethality. These humanized *Mut*^-/-^;*MUT*^2h^ mice represent a valuable tool for studying the long-term effects of elevated methylmalonic acid levels and evaluating potential therapeutic strategies [37, 38]. In addition, researchers have developed tissue-specific murine models expressing *MMUT* under liver-specific (*Mut*^-/-^*;Tg*^INS-Alb-Mut^) or muscle-specifc (*Mut*^-/-^*;Tg*^INS-MCK-Mut^) promoters. These models facilitate the study of tissue-specific contributions to MMA pathophysiology and treatment outcomes [39–44].

### *MMUT* knock-in mouse models

In addition to knockout models, researchers have created knock-in mice that mimic specific *MMUT* mutation identified in the patient. For example, Forny et al. introduced the human *MMUT c.2009T > A* (p.M700K) mutation into embryonic stem cells to generate a knock-in (ki) mouse line (*Mut*^ki/ki^) [45]. To assess the effect of genetic dosage on the phenotype and to create a more severe disease model, the ki mutation was combined with a knockout (ko) allele. Both *Mut*^ki/ki^ and *Mut*^ko/ki^ mice exhibited extended survival but showed slow growth, while *Mut*^ko/ki^ mice demonstrated lower MCM activity and higher methylmalonic acid levels. When challenged with a high-protein diet, both models displayed increased blood ammonia and weight loss; however, only *Mut*^ki/ki^ mice responded to cobalamin treatment during metabolic crises, demonstrating gene dosage dependence [45]. These model mice are suitable for studying the chronic clinical manifestations and pathophysiological mechanisms of *mut-*type MMA due to their long survival [46–48].

Another knock-in model, *Mut*^-/-^*;Tg*^INS-CBA-G715V^, was created to replicate the G717V mutation commonly observed in MMA patients [49]. These mice showed reduced MCM activity across all tissues and moderately increased plasma methylmalonic acid levels, mimicking the phenotype of MMA patients with the G717V mutation. Importantly, *Mut*^-/-^*;Tg*^INS-CBA-G715V^ mice exhibited normal lifespans and survived high-protein diet challenges, making them suitable for drug intervention studies [49–52].

More recently, Schneller et al. used CRISPR-Cas9 genome editing to introduce *mut*^0^ (MMUT p.R108C) or *mut*^-^ (MMUT p.G717V) missense mutations into mice [53]. Mice homozygous (*Mmut*^p.R106C/p.R106C^) for the human MMUT p.R108C mutation exhibited a survival rate of less than 10% by day 57, with most deaths occurring within the first two weeks. However, postnatal treatment with an AAV serotype 9 vector expressing a human codon-optimized *MMUT* rescued most of the affected mice, enabling normal survival. Conversely, *Mmut*^p.G715V/p.G715V^ demonstrated no significant mortality by day 111, aligning closely with the *Mut*^-/-^*;Tg*^INS-CBA-G715V^ mice [49].

### *cblC*-type mouse models

To explore the role of MMACHC during mouse embryonic development, Moreno-Garcia et al. developed a *cblC* mouse model with a gene-trap insertion in intron 1 of the *MMACHC* gene (*Mmachc*^Gt^(*AZ0348)Wtsi*). They demonstrated that *Mmachc*^Gt/+^ mice exhibited a 50% reduction in MMACHC protein levels and increased plasma homocysteine and methylmalonic acid concentrations. Notably, no *Mmachc*^Gt/Gt^ embryos were found beyond embryonic day (E) 3.5, indicating an essential role for MMACHC in early embryonic development [54].

In 2020, Chern et al. described three distinct mouse models related to MMACHC [55]. The first model, a targeted allele of *MMACHC* (*Mmachc*^tm1.1^), replaces exons 3 and 4 with a lacZ cassette. This alteration produces a truncated protein containing only the first 92 amino acids of the full 279. Unlike the preimplantation lethality observed in *Mmachc*^GT/GT^ mice [54], *Mmachc*^tm1.1/tm1.1^ mice survive until approximately E9.5–E15.5. These mice exhibited novel phenotypes, including abnormalities resembling those observed in patients with *cblC*-type MMA. Given the phenotypic severity and embryonic lethality in both *Mmachc*^GT/GT^ and *Mmachc*^tm1.1/tm1.1^ mice, Chern et al. further generated a conditional knockout mouse (*Mmachc*^flox/flox^) to enable temporal- and tissue-specific deletion of *MMACHC.* For example, the transgenic mouse (*Mmachc*^flox/flox^*;Pax6-Cre*) specifically lacks *MMACHC* expression in peripheral retinal cells [56]. When crossed with *E2A-Cre*^+/tg^ germline deleter mice，the offspring (*E2A-Cre*^+/tg^*;Mmachc*^+/flox^) were viable, but *E2A-Cre*^+/tg^*;Mmachc*^flox/flox^ embryos did not survive beyond E15.5, consistent with findings from *Mmachc*^GT/GT^ and *Mmachc*^tm1.1/tm1.1^ mice.

To investigate the functional role of *MMACHC* under gain-of-function conditions, Chern et al. developed a transgenic mouse model (*Mmachc-OE*^+/tg^) with widespread overexpression of the *MMACHC* gene [55]. Preliminary findings indicated that overexpression alleviated the phenotypes observed in *Mmachc*^tm1.1/tm1.1^ mice without causing deleterious effects, suggesting a potential therapeutic role for enhanced *MMACHC* expression.

In recent years, researchers have also employed CRISPR/Cas9 technology to introduce *MMACHC* mutations commonly observed in humans into mice. For example, the *c.609 G > A* mutation, the most prevalent *MMACHC* mutation in China, is a nonsense mutation that results in a premature stop codon (p.W203X). Homozygous mice carrying this mutation exhibited significantly elevated C3 levels within 24 hours of birth and died within 72 hours, closely mirroring the severe clinical presentation of pediatric patients with this mutation, who rarely survive beyond the first week of life [57]. Another clinically significant mutation, *c.80A > G* (p.Q27R), is a common missense mutation associated with late-onset *cblC*-type MMA. This mutation is often linked to various secondary conditions, including kidney disease, congenital heart defects, hypertension, pulmonary artery hypertension, and diffuse lung disease [58, 59]. In 2023, Wang et al. successfully generated a *cblC*-type mouse model with the *MMACHC c.80A > G* (p.Q27R) mutation using CRISPR/Cas9 technology [60]. These mice exhibited long survival times, allowing researchers to simulate blood metabolic changes and study biochemical and pathological alterations characteristic of *cblC*-type MMA. By 12 weeks, homozygous mutant mice displayed mild neurological symptoms, such as glial cell proliferation, neuronal vacuoles, and cortical vessel congestion. However, the association between these findings and cognitive function remains unclear.

In addition to directly targeting the *MMACHC* gene, researchers have also explored indirect mechanisms for inducing *cblC*-like phenotypes. For example, interference with upstream transcriptional regulators of *MMACHC*, such as Host Cell Factor C1 (HCF1) and THAP Domain Containing 11 (THAP11), reduces *MMACHC* mRNA expression during development, resulting in phenotypes resembling *cblC*-type MMA [61].

## Zebrafish MMA models

In recent decades, zebrafish have emerged as a valuable model for studying rare inherited metabolic disorders and developing novel therapeutic strategies. Compared to other animal models and in vitro cell cultures, zebrafish present several distinct advantages. First, the evolutionary conservation of genes and proteins between zebrafish and humans is remarkably high, enabling the study of genes implicated in human diseases. Additionally, zebrafish embryos develop rapidly, allowing initial metabolic screenings to be performed within 24 hours post-fertilization (HPF). Their transparency during early development further facilitates real-time visualization of organogenesis and cellular processes. Moreover, zebrafish are highly amenable to genetic manipulation through techniques such as morpholino knockdown, TALEN, CRISPR/Cas9, and transgenic modifications, making them an exceptionally powerful model for exploring inherited disorders, including MMA [62]. Although zebrafish are less commonly utilized in MMA research compared to rodent models, they offer unique benefits for investigating developmental aspects of the disease and conducting high-throughput drug screening.

To overcome the limitations posed by the lethality of *MMUT* knockout mouse models, *MMUT*-deficient zebrafish were generated using CRISPR/Cas9 technology [47]. These zebrafish exhibited key MMA phenotypes, including liver and kidney mitochondriopathy, behavioral abnormalities, excessive mortality, and increased mitochondrial oxidative stress. The high larval mortality rate was partially mitigated by a low-protein diet, which is currently a standard therapeutic intervention for patients with MMA. This zebrafish model also facilitated the identification of potential therapeutic targets predicted through drug-disease network-based computational modeling [63]. For example, the mitochondrial antioxidant Mito-Q, has been investigated in preclinical models of lysosomal storage disorders for its ability to restore mitochondrial and lysosomal function [64, 65], and was shown to alleviate oxidative stress and reduce disease severity in *MMUT*-deficient zebrafish. Furthermore, this model provided mechanistic insights into *MMUT* deficiency-induced MMA by revealing that mutant *MMUT* disrupts the PINK1-mediated translocation of PRKN/Parkin to damaged mitochondria, impairing their clearance via macroautophagy/autophagy-lysosome degradation pathways [47].

Recently, Sloan et al. developed a zebrafish germline mutant for *cblC* (*hg13* allele). This mutant exhibited hallmark symptoms of MMA, including elevated methylmalonic acid levels, retinopathy, and juvenile mortality [66]. Several small molecules, including hydroxocobalamin and methylcobalamin, were effective in ameliorating MMA-associated phenotypes in this model, underscoring its utility for evaluating potential therapeutic strategies. In addition to metabolic abnormalities, the *hg13* allele displayed craniofacial defects, such as elongated Meckel’s cartilage, increased spacing in the palatoquadrate structures, atypical chondrocyte organization, and excessive cellular connections in the hyosymplectic cartilage. These defects in chondrocyte development were reversed by treating the zebrafish with mRNA encoding either the wild-type human MMACHC protein or the patient-derived p.Gly147Asp variant, which is located within the cobalamin-binding domain of MMACHC [67]. Interestingly, these findings suggest that regions outside the cobalamin-binding domain are also critical for normal craniofacial development.

## Conclusions and prospects

MMA is a rare genetic metabolic disorder caused by mutations in various genes, resulting in diverse subtypes with complex etiologies and ambiguous clinical symptoms. Research on MMA pathogenesis and the progression of neurological damage has been hindered by the rarity of the disease and ethical limitations on collecting patient tissue samples. The development of appropriate animal models for MMA plays a critical role in addressing these challenges, as such models can simulate patient symptoms and enable the study of the disease’s natural history, including behavioral characteristics, metabolic byproducts, and tissue pathology. These models provide opportunities to correlate specific mutation sites with phenotypes, analyze the pathogenesis of distinct gene mutations, improve our understanding of MMA, and facilitate the development of novel therapeutic approaches. However, most currently available MMA animal models result in embryonic lethality, significantly restricting the study window and limiting the observable phenotypes. Consequently, there is an urgent need to create MMA animal models with extended survival times to enable more comprehensive research on disease mechanisms. Targeting high-frequency mutations found in late-onset MMA patients and utilizing CRISPR/Cas9 genome-editing technology to generate mice with these mutations could enhance the possibility of developing models with longer survival times, thereby improving the simulation of disease onset and progression. Moreover, although cobalamin is widely used in the clinical management of MMA, its responsiveness and therapeutic efficacy have been insufficiently characterized in animal models. This underscores the need for future studies to establish dose–response relationships and define therapeutic windows in relevant cobalamin-treated MMA animal models.

Another limitation of current MMA animal models is their reliance on systemic gene interference, which complicates the determination of whether specific clinical symptoms result from systemic effects or localized changes in organs or tissues due to genetic mutations. To address this issue, researchers can hybridize conditional knockout mice with CRE or CRE-ER mice carrying tissue- or cell-specific promoters, or alternatively, use localized injections of viral vectors encoding CRE enzymes. These approaches enable precise spatial and temporal manipulation of MMA pathogenesis genes, aligning the experimental model with specific research objectives.

Large animal models, including pigs and non-human primates, offer a closer approximation to human physiology, particularly in terms of organ size, metabolic processes, and disease progression [68]. Recent advances in CRISPR-Cas9 genome-editing technology have enabled the development of porcine models that exhibit MMA-like phenotypes [69]. These models are especially valuable for evaluating the safety and efficacy of organ-specific therapies, such as liver and kidney transplantation, which are challenging to study in smaller animal models due to anatomical and physiological differences [70].

Advances in organ-on-chip technologies and induced pluripotent stem cell (iPSC)-derived organoids could serve as valuable complements to animal models, offering innovative platforms for drug discovery and mechanistic investigations. Additionally, integrating omics technologies, such as genomics, transcriptomics, and metabolomics, with animal research may provide deeper insights into disease mechanisms and identify novel therapeutic targets.

In summary, animal models have been instrumental in advancing our understanding of MMA and its treatment. Despite their limitations, they provide a crucial foundation for translational research and therapeutic innovation. By refining existing models and embracing new technologies, future studies can bridge the gap between preclinical research and clinical applications, ultimately improving outcomes for MMA patients.

## Data Availability

Not applicable.
